# Negative Curvature Hollow Core Fiber Based All-Fiber Interferometer and Its Sensing Applications to Temperature and Strain

**DOI:** 10.3390/s20174763

**Published:** 2020-08-23

**Authors:** Dejun Liu, Wei Li, Qiang Wu, Haoyu Zhao, Fengzi Ling, Ke Tian, Changyu Shen, Fangfang Wei, Wei Han, Gerald Farrell, Yuliya Semenova, Pengfei Wang

**Affiliations:** 1 Key Laboratory of Optoelectronic Devices and Systems of Ministry of Education and Guangdong Province, College of Physics and Optoelectronic Engineering, Shenzhen University, Shenzhen 518060, China; dejun.liu@szu.edu.cn (D.L.); 1810285019@email.szu.edu.cn (W.L.); fzling@szu.edu.cn (F.L.); 2 Key Laboratory of Nondestructive Test (Ministry of Education), Nanchang Hangkong University, Nanchang 330063, China; qiang.wu@northumbria.ac.uk; 3 Department of Mathematics, Physics and Electrical Engineering, Northumbria University, Newcastle Upon Tyne NE1 8ST, UK; 4 Technical Center, Sichuan Changhong Electric Co., Ltd., Mianyang 621000, China; haoyu.zhao@changhong.com; 5 Key Lab of In-fiber Integrated Optics, Ministry Education of China, Harbin Engineering University, Harbin 150001, China; ketian@hrbeu.edu.cn; 6 Institute of Optoelectronic Technology, China Jiliang University, Hangzhou 310018, China; shenchangyu@cjlu.edu.cn; 7 Photonics Research Centre, Technological University Dublin, Kevin Street, Dublin 8, Ireland; fangfang.wei@tudublin.ie (F.W.); D14125980@mytudublin.ie (W.H.); gerald.farrell@tudublin.ie (G.F.); yuliya.semenova@tudublin.ie (Y.S.)

**Keywords:** fiber interferometers, negative curvature hollow core fiber, optical fiber sensors, simultaneous measurement, temperature, strain

## Abstract

Negative curvature hollow core fiber (NCHCF) is a promising candidate for sensing applications; however, research on NCHCF based fiber sensors starts only in the recent two years. In this work, an all-fiber interferometer based on an NCHCF structure is proposed for the first time. The interferometer was fabricated by simple fusion splicing of a short section of an NCHCF between two singlemode fibers (SMFs). Both simulation and experimental results show that multiple modes and modal interferences are excited within the NCHCF structure. Periodic transmission dips with high spectral extinction ratio (up to 30 dB) and wide free spectral range (FSR) are produced, which is mainly introduced by the modes coupling between HE_11_ and HE_12_. A small portion of light guiding by means of Anti-resonant reflecting optical waveguide (ARROW) mechanism is also observed. The transmission dips, resulting from multimode interferences (MMI) and ARROW effect have a big difference in sensitivities to strain and temperature, thus making it possible to monitor these two parameters with a single sensor head by using a characteristic matrix approach. In addition, the proposed sensor structure is experimentally proven to have a good reproducibility.

## 1. Introduction

Optical fiber interferometric sensors have been widely investigated and employed for monitoring the changes of a wide range of physical and bio-chemical parameters, with their inherent advantages of simple structure, ease of fabrication, high sensitivity, immunity to electromagnetic interference and ability to work in harsh environments and to operate remotely [1,2]. To date, a number of fiber interferometers have been proposed based on different types of fibers, such as multimode fibers (MMFs) [3], coreless fibers [4], thin core fibers [5], multicore fibers [6], and photonic crystal fibers (PCFs) [2,7,8,9]. Among them, PCFs with micro-structured air holes within the silica matrix allow for greater flexibility in the design, and hence PCF-based interferometers have been intensively investigated. However, interferometers based on PCFs suffer from relatively high loss and poor reproducibility, since the small air cores in PCFs are easy to collapse during fusion splicing, and both the splice power and arc duration are found to have significant influence on the final transmission spectrum of the PCF-based structure [2,10].

In 2011, negative curvature hollow core fiber (NCHCF) was first proposed by Pryamikov et al. [11] with a significantly lower transmission loss compared to other hollow core PCFs. The proposed waveguide regime was characterized with a negative curvature of the core-cladding interface. In less than 10 years since its first appearance, the NCHCF attracted tremendous attention for various applications, such as high power/ultrafast laser delivery and other laser-related applications due to its advantages of large bandwidth, reduced transmission loss, simple structure and easy fabrication [12,13]. However, while NCHCF is a promising candidate for sensing applications, research on NCHCF based fiber sensors only began in the recent two years. For example, Wei et al. proposed and investigated theoretically a temperature sensor based on a liquid filled NCHCF [14]; Silva et al. theoretically demonstrated the possibility of monitoring the concentration of three toxic gases utilizing a NCHCF [15]; Cubillas et al. reported that it was possible to monitor chemical reactions with very small quantum yields by using a liquid filled NCHCF [16]. Yao et al. proposed a photothermal CO sensor based on a hollow-core negative curvature fiber combined with a free space Mach–Zehnder interferometer [17]. Qiao et al. designed an optical fiber sensing platform for the ultra-sensitive detection of bisphenol A (BPA) based on a black phosphorus integrated NCHCF [18]. It should be noted that the above studies are either limited to theoretical simulations or involve sensor structures that are not entirely all-fiber.

In this work, an all-fiber NCHCF based interferometer is proposed for the first time to the best of our knowledge, by splicing a short section of a NCHCF between two singlemode fibers (SMFs). The NCHCF structure has a relatively simple configuration, its transmission spectrum exhibits a high extinction ratio (up to 30 dB) periodic dips and a wide and adjustable free spectral range (FSR), dependent on the length of NCHCF, which allows it to be used both as a high quality comb filter and as a sensor. Both theoretical analysis and experimental results demonstrate that multiple modes and modal interferences exist within the NCHCF while a small portion of light guiding through an anti-resonant reflecting optical waveguide (ARROW) mechanism is also observed. Since the dips introduced by multimode interferences (MMI) and ARROW principle have different sensitivities to strain and temperature, it could be used as a dual-parameter sensor for the simultaneous measurement of strain and temperature. In addition, it is demonstrated that the proposed NCHCF structure has a good reproducibility.

## 2. Experimental Setup

**Figure 1:**
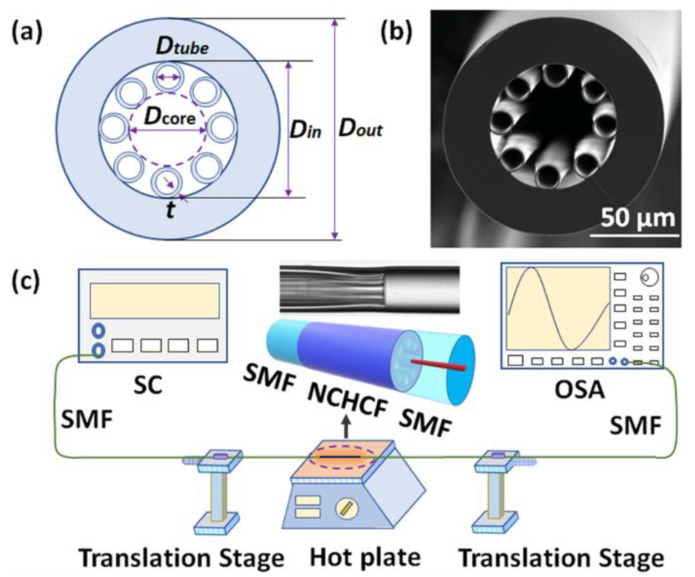
(**a**) Schematic diagram and (**b**) SEM image of the negative curvature hollow core fiber (NCHCF); (**c**) schematic diagram of the experimental setup for strain and temperature measurements. Inset microscope image shows the connecting point between singlemode fibers (SMF) and NCHCF after one arc splice.

In our experiment, an NCHCF with eight standalone thin silica cladding capillary tubes (purchased from Changyingtong Wuhan Co. Ltd., Wuhan, China) was used. Figure 1a,b illustrate a schematic diagram and a scanning electron microscope (SEM) image of the cross-section of the NCHCF. In Figure 1a, the silica glass material is marked in light blue color while the air holes are represented as white-colored regions. The NCHCF has an outer diameter (*D_out_*) of 125 μm, an inner diameter (*D_in_*) of 76 μm, each thin silica capillary tube has an inner diameter (*D_tube_*) of 14 μm, a silica strut thickness (*t*) of 1.4 μm and a core diameter (*D_core_*) of approximately 45.2 μm.

Figure 1c illustrates a schematic diagram of the experimental setup for temperature and strain measurements. The NCHCF-based modal interferometer was fabricated by fusion splicing a short section of NCHCF between two SMFs using a Fujikura 62S+ fusion splicer (Fujikura (China) Co., Ltd., Shanghai, China). The splicing parameters were carefully chosen to obtain a good transmission spectrum with low loss, high visibility and good reproducibility. The arc power was set to STD-75 bit, the arc time was 1 s. A microscope image of the splice is shown in the inset figure, as an example. It is found that the thin silica tubes in NCHCF were collapsed slightly close to the splice point. Then, two ends of the SMFs were fixed on two manual translation stages to realize strain measurement. A temperature test was carried out by placing the sensor structure on a controllable hot plate. Light from a broadband supercontinuum source (SC, SC-YSL, YSL Photonics, Wuhan, China) was launched into the NCHCF based structure and the transmitted light was interrogated by an optical spectrum analyzer (OSA, Yokogawa AQ6370D, YOKOGAWA, Tokyo, Japan). The NCHCF structure was connected to the SC and OSA through SMFs.

## 3. Theoretical Analysis

**Figure 2:**
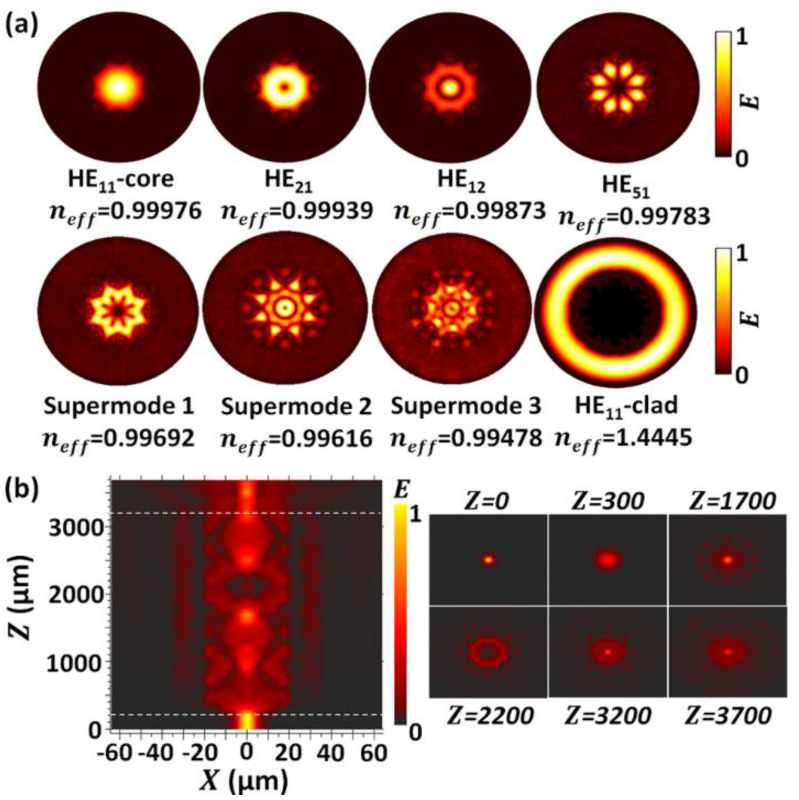
(**a**) Examples of different mode profiles (normalized) within the NCHCF, and their corresponding effective refractive indices, simulated by an finite-element method (FEM).; (**b**) Energy distributions in the XZ plane along the SMF-NCHCF-SMF length (lengths of the input and output SMF, and NCHCF are 200 μm, 500 μm and 3000 μm, respectively) and the mode profile evolution at the cross-section at different fiber structure lengths, simulated by a beam propagation method (BPM).

Since the refractive index (RI) of the air core is smaller than that of the silica cladding wall, light propagating in a NCHCF is not confined due to total internal reflection as in conventional fibers. Its guiding mechanism can be explained with the “inhibited coupling” model where the coupling between the fundamental core mode and the cladding modes are inhibited by reduced modes overlap and wave number mismatch [13,19]. In this work, we calculated the mode field distributions and their corresponding effective refractive indices (*n_eff_*) with a finite-element method (FEM). Throughout the simulation, the input light wavelength is set to 1300 nm, the refractive indices (RIs) of the core and cladding of the SMF are 1.4519 and 1.4469 and the RIs of the silica cladding of NCHCF and air are 1.4469 and 1.0, respectively. The RI of the silica cladding is calculated from the Sellmeier equation [20]. Figure 2a shows examples of the simulation results of different modes in an NCHCF, including the fundamental air core mode, high order air core modes, supermodes (hybrid modes between air core modes and capillary air cladding modes) and silica cladding modes. It is found that the air cladding modes propagating within the capillary tubes have slightly lower *n_eff_* than that of the fundamental air core mode (HE_11_), thus high order air core modes are easier to couple into the capillary air cladding, and those modes propagating in the air core and capillary air cladding interfere with each other, producing the supermodes. In the NCHCF, most of the light is confined in the air core and capillary tubes, and only a very small portion of light is coupled into the outer silica cladding.

When a short section of an NCHCF is sandwiched between two SMFs, the NCHCF acts as a multimode fiber, and multiple mode interferences (MMI) occur within the NCHCF section, resulting in the formation of a modal interferometer. Using the FEM to simulate the light propagation in a relatively long 3-dimensional NCHCF structure is computationally very intensive, hence in this work the beam propagation method (BPM) has been used. Figure 2b shows the energy distribution along the SMF-NCHCF-SMF length and the mode field evolution at different cross-sections along the NCHCF length. It is evident that multimode interferences occur within the NCHCF, and light propagating in the air core is coupled into the capillary air core while a very small portion of light penetrates to the outer silica cladding.

The MMI inside the proposed NCHCF structure can be understood with a “two-beam optical interference model”, where the total light intensity (*I*) for two interfering modes can be expressed by [21]:

(1) $$I=I_{1}+I_{2}+2\sqrt{I_{1}I_{2}}\cos\varphi ,$$

where *I*_1_ and *I*_2_ are light intensities of the two modes involved in the interference, *φ* is the phase difference between the two modes. Assuming the incident light has a wavelength of λ, ∆*n_eff_* is the difference between the effective refractive indices of the two interfering modes and *L* is the length of the sensor head, then:

(2) $$\varphi =2\pi \Delta n_{eff}L/\lambda ,$$

(3) $$\Delta n_{eff}=n_{eff}^{2}-n_{eff}^{1},$$

Destructive interference occurs when the phase difference is an odd multiple of *π*, the resonant dips (*λ_dip_*) shown in the transmission spectrum are thus can be derived as:

(4) $$\lambda_{dip}=2\Delta n_{eff}L/(2m+1),$$

The corresponding FSR between two adjacent transmission dips can be described by the formula:

(5) $$FSR=\frac{\lambda_{1}\lambda_{2}}{\Delta n_{eff}L},$$

where *λ*_1_ and *λ*_2_ are the central wavelengths of the two adjacent transmission dips. As can be seen from Equations (4) and (5), the positions of the transmission dips and their corresponding FSRs are dependent on the difference of effective RIs of the interfering modes and the length of the NCHCF. Since the RIs of the fiber core, cladding and fiber length are dependent on the surrounding temperature and strain applied to the fiber, the proposed NCHCF-based fiber interferometer can be easily employed as a temperature (*T*) and stain (*ε*) sensor by measuring the wavelength shifts of the transmission dips. The temperature and strain sensitivities can be obtained from Equation (4), as in [22]:

(6) $$\frac{\partial \lambda_{dip}}{\partial T}=\left[\frac{1}{\Delta n_{eff}}\frac{\partial \Delta n_{eff}}{\partial T}+\frac{1}{L}\frac{\partial L}{\partial T}\right]\lambda_{dip},$$

(7) $$\frac{\partial \lambda_{dip}}{\partial \varepsilon }=\left[\frac{1}{\Delta n_{eff}}\frac{\partial \Delta n_{eff}}{\partial \varepsilon }+1\right]\lambda_{dip},$$

where *ε = dL/L* indicates the applied strain. It can be seen from Equations (6) and (7) that the temperature and strain sensitivities are determined by ∆*n_eff_*, *L* and their corresponding changes towards strain and temperature variations, and the selected *λ_dip_*.

## 4. Results and Discussion

### 4.1. Spectral Response in Air

**Figure 3:**
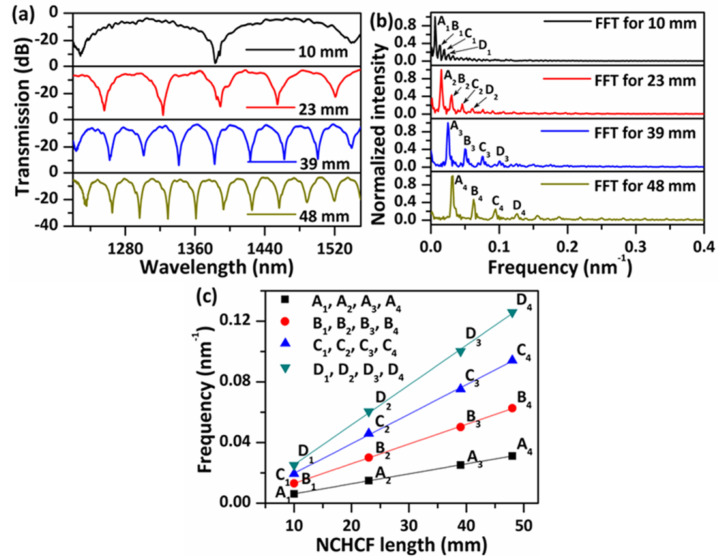
(**a**) Measured transmission spectra and (**b**) fast Fourier transform (FFT) spatial frequency spectra corresponding to those in (**a**), and (**c**) spatial frequency variations versus the NCHCF length.

In order to gain a better understanding of the impact of the length of the NCHCF section, prior to performing the temperature and strain tests, the transmission spectral responses in air of the proposed sensor structures with different lengths of the NCHCF were firstly measured and analyzed, and some of the examples are illustrated in Figure 3a. Series of periodic transmission dips with large extinction ratios (up to ~30 dB) and wide FSR have been observed for all samples over a wide wavelength range from 1220 nm to 1550 nm. The FSR is much bigger than previous reported PCFs based interferometers with the same sensor lengths [7,23,24]. As the length of the NCHCF increases from ~10 mm to ~48 mm, the measured spectral FSR decreases. This is a typical feature of such interferometers, agreeing well with Equation (5). The insertion loss of the structure is approximately 3.5 dB, and no obvious changes in the insertion loss are observed with the increase in the NCHCF length, demonstrating its much lower transmission loss than that of an annular capillary fiber (hollow core fiber) as reported in our previous work [25]. It is well known, that large FSR and low transmission loss enable a wide measurement range and the possibility of cascading of more fiber sensors within a single system, while high extinction ratio dips can facilitate higher sensitivity in case of intensity demodulation, and higher spectral resolution in case of demodulation in the wavelength domain, hence the NCHCF structure shows promising sensing applications. Figure 3b shows the fast Fourier transform (FFT) spatial frequency spectra corresponding to the measured transmission spectra in Figure 3a. Several spatial frequency peaks are observed, which confirms that multiple modes are involved in the interference fringe patterns. Four main peaks in each of the spatial frequency spectra for the different sensor lengths are labeled as A_1_, A_2_, A_3_, A_4_, B_1_, B_2_, B_3_, B_4_, C_1_, C_2_, C_3_, C_4_, D_1_, D_2_, D_3_, D_4_, respectively. The relationship between the spatial frequencies (corresponding to the above frequency peaks) and the sensor length is plotted in Figure 3c. In the figure, the straight lines are the linear fittings for the data obtained from Figure 3b. As one can see from the figure, the spatial frequency increases linearly with the sensor length, indicating that interferences in samples with different sensor lengths are created by the same modes. The result agrees well with the theoretical prediction, where the spatial frequency (*ξ*) can be expressed as [26]:

(8) $$\xi =\frac{\Delta m_{eff}L}{\lambda_{dip}^{2}},$$

where ∆*m_eff_* is the differential modal group index. The slope of the fitted curve in Figure 3c is determined by the difference in the effective group index for the interfering modes.

### 4.2. Temperature and Strain Measurement

**Figure 4:**
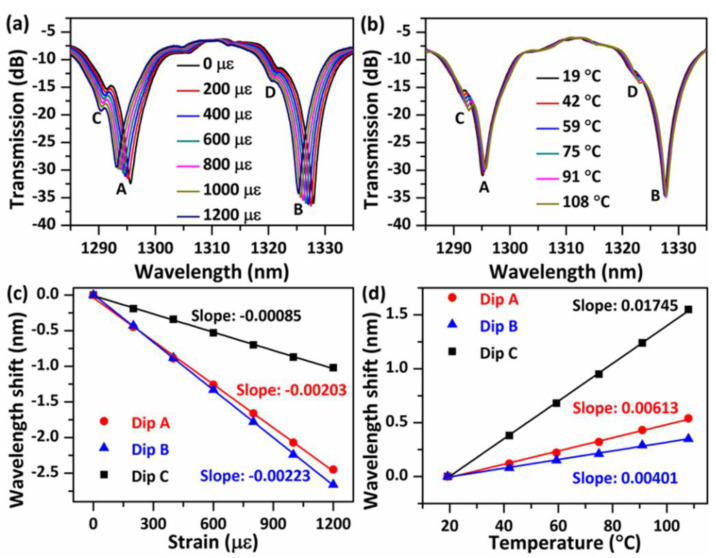
Measured spectral responses of S-48 versus (**a**) strain in the range from 0 to 1200 με and (**b**) temperature in the range from 19 °C to 108 °C, and the corresponding wavelength shifts of dips A, B, and C in relation to (**c**) strain and (**d**) temperature. The straight lines are linear fits for the measured data.

A sensor sample with a NCHCF length of ~48 mm (denoted as S-48) is chosen for the experimental demonstration of its sensing properties for strain and temperature. The sample with a longer length of NCHCF is used because it possesses a higher quality (Q, defined as the ratio of a dip’s central wavelength to its full width at half-maximum bandwidth) factor and a “cleaner (smoother)” spectrum, and hence gives a more accurate test result and the potential for a higher sensitivity in an actual sensing system. Examples of the measured spectral responses from 1285 nm to 1335 nm under various strains and temperatures are shown in Figure 4a,b. The spectrum moves monotonically towards shorter wavelengths as the strain increases, while a red shift is observed with the increase in temperature. Three transmission dips (dips A, B, and C) with relatively large strengths were employed to calculate their wavelength shifts versus strain and temperature, the corresponding results are presented in Figure 4c,d. In our experiment, a blue wavelength shift is referred to as a negative wavelength shift, while a red wavelength shift is referred to as a positive wavelength shift. The results show that, for all three selected dips, the measured wavelength shifts linearly increase with strain and temperature. The measured strain sensitivities for dips A, B and C are −2.03 pm/με, −2.23 pm/με, and −0.85 pm/με, respectively. The measured temperature sensitivities for dips A, B and C are 6.13 pm/°C, 4.01 pm/°C, and 17.45 pm/°C, respectively. The maximum temperature and stain sensitivities are better than that of a typical FBG sensor and are compatible with that of many other PCFs-based sensors and multi-parameter sensors [27,28,29,30].

**Figure 5:**
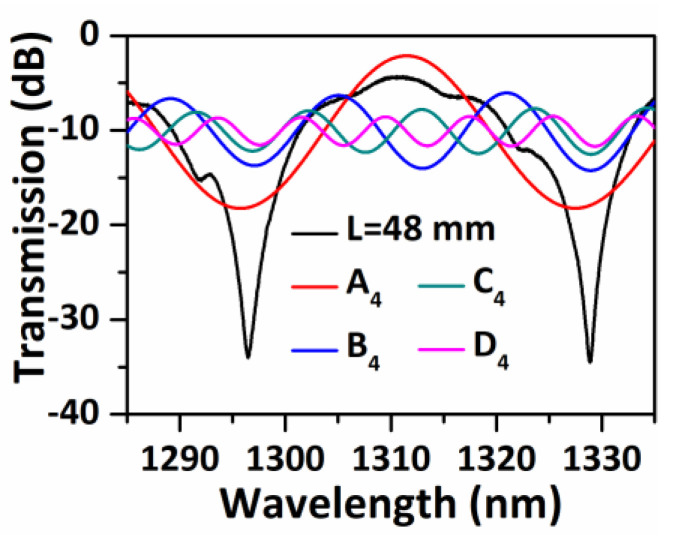
Measured spectral responses of S-48 and fast Fourier transform (FFT) band pass filter recovered transmission spectra corresponding to different frequencies of A_4_, B_4_, C_4_, D_4_.

To determine the origin of the dips, the band pass filter method is applied in FFT, the transmission spectra of the sample S-48 are recovered for each corresponding frequency (A_4_, B_4_, C_4_, D_4_) as shown in Figure 5. It is evident that the spectrum corresponding to frequency A_4_ is the main contribution to dips A and B. It is known that ∆*n_eff_* can be calculated with the following equation [31]:

(9) $$\Delta n_{eff}=\frac{\lambda^{2}}{\Delta \lambda L},$$

where *λ* and ∆*λ* are the central wavelength and FSR between two dips. Given that the FSR of the transmission spectral of frequency A_4_ is 31.73 nm at 1300 nm, then ∆*n_eff_* is calculated to be 1.13 × 10^−3^, which is very close to the simulated ∆*n_eff_* (1.03 × 10^−3^) between HE_11_ and HE_12_ modes at 1300 nm, it is thus concluded that the modes coupling between HE_11_ and HE_12_ are the dominant modal interferences, and produce the large extinction ratios dips in transmission.

**Figure 6:**
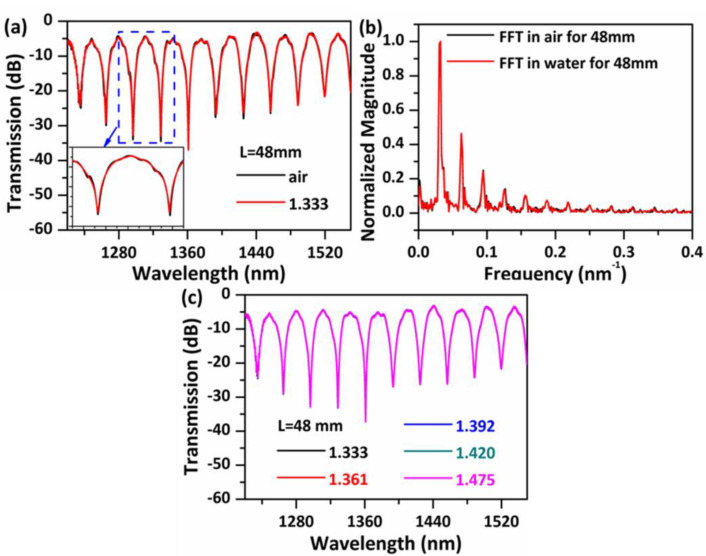
(**a**) Measured transmission spectra for S-48 in air and water (RI = 1.333) and (**b**) their corresponding FFT spatial frequency spectra. (**c**) Measured transmission spectra for S-48 in liquids with different refractive indices (RI).

For completeness, the sensitivity of the sensor to the RI of surrounding environment was also checked. Initially, the sensor’s performance for a significant step change in the external RI was investigated as shown in Figure 6. Figure 6a compares the transmission spectra in air and water, the major dips remain almost unchanged in the presence of a step change in the RI. Further FFT analysis (Figure 6b) found that the main peaks in the spatial frequency spectra change only slightly, but some smaller peaks located at relatively high frequencies disappear and correspond to the spatial frequencies of the disappeared transmission dips in Figure 6a. This is reasonable, since modes HE_11_ and HE_12_ are well confined inside the central hollow core and they are isolated from the outer environment.

Subsequently, further investigations of the transmission spectra for a range of RI liquids were performed as presented in Figure 6c, and no changes were observed in dip positions and dip strengths, which indicates that these strong interference dips are independent on the surrounding RI. This independence from the surrounding RI provides the sensor with a very useful advantage in that it can be used in a wider range of applications, compared to other PCF-based modal interferometers [32,33]. However, some small dips disappear when the sensor head is covered with water, examples including dips C and D as shown in Figure 4. Note that in our previous work [25,34], we have demonstrated that an annular capillary fiber with an inner diameter of 30 μm has a temperature and strain sensitivity of circa 18 pm/°C and 0.66 pm/με, while the resonant transmission dips produced by the ARROW effect disappear when the annular capillary fiber is covered with water. In this work, dip C (or dip D) shows very similar temperature and strain sensitivities and a similar performance when the NCHCF is covered with water; hence, it is reasonable to deduce that a small portion of light is guided through ARROW effect. A small portion of light coupling into the thin capillary cladding leaks into the outer silica cladding through the connection nodes, if the resonance condition is satisfied, ARROW guidance is excited. The FSR of the resonant dips produced by the ARROW principle can be calculated as [35]:

(10) $$\Delta \lambda =\frac{\lambda_{m}\lambda_{m+1}}{2d}\sqrt{n_{2}^{2}-n_{1}^{2}},$$

where *λ_m_* and *λ_m+_*_1_ are the central wavelengths of adjacent resonant dips, *n*_1_ and *n*_2_ are the refractive indices of silica cladding and air, *d* is the outer silica cladding thickness. The calculated FSR is 32.8 nm which matches well with the experimental measured FSR of 30.4 nm between dips C and D. Silica material has a relatively high thermo-optical coefficient, and hence a higher temperature sensitivity. However, variations of the central wavelengths of the dips produced by the ARROW effect are independent of the change of sensor head length [25], thus they have lower strain sensitivities than those of dips produced by MMI. It is also noted that much higher temperature sensitivities could be potentially achieved by the functionalization of the NCHCF with liquids or other materials, which is under investigation.

Since dips A (or B) and C have a big difference in sensitivity to strain and temperature, the simultaneous measurement of strain and temperature could be potentially realized by establishing a characteristic matrix equation:

(11) $$\left[\begin{array}{l} \Delta T\\ \Delta \varepsilon \end{array}\right]={\left[\begin{array}{ll} -2.03 & -0.85\\ 6.13 & \;17.45 \end{array}\right]}^{-1}\left[\begin{array}{l} \Delta \lambda_{A}\\ \Delta \lambda_{C} \end{array}\right],$$

where ∆*λ_A_* and ∆*λ_C_* are wavelength shifts of dips A and C, and ∆*T* and ∆*ε* are temperature and strain variations.

## 5. Reproducibility

**Figure 7:**
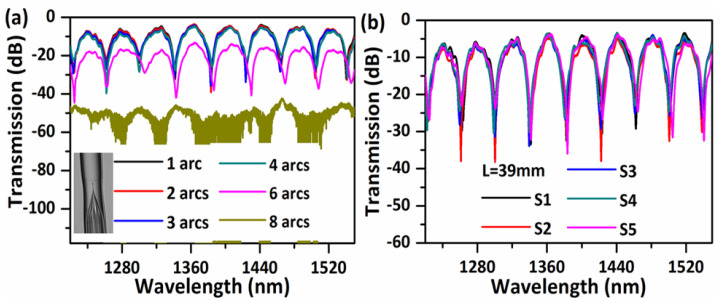
(**a**) Measured transmission spectra of five reproduced sensor samples with a NCHCF length of 39 mm; (**b**) Measured transmission spectra with different number of arcs performed on the splice point between SMF and NCHCF.

The influence of the fusion splicing parameters including arc power and arc time on the transmission spectrum was also investigated, as shown in Figure 7a. In the experiment, the arc was performed and repeated on the splice point for a number of times while the arc power (−75 bit) and arc time (1 s) were constant. As can be seen from Figure 7a, the insertion loss of the sensor becomes larger with an increase in the number of arcs applied in sequence. This is reasonable, since multiple arcs result in a more significant collapse of the hollow core and thus more light energy is coupled into the cladding and leaks out to the outer air. However, it is found that the spectra obtained for the samples fabricated with less than four arcs match well with each other with only slight variations in dips’ strength and position, which demonstrates that the sensor has a relatively high tolerance to variations in the splicing parameters and hence a good reproducibility. The sensor starts to show a significant loss in its transmission after six arcs, but the main features of the transmission spectrum remain unchanged. After eight subsequent arcs, the large air core collapsed completely and a sharp angle was created at the air core/silica cladding interface (a microscope image is shown in the inset figure of Figure 7a, most of the light leaks out to the surrounding environment. The reproducibility of the sensor was demonstrated with a sensor with an NCHCF length of ~39 mm. Figure 7b presents the transmission spectra of five different samples (denoted as S1, S2, S3, S4, and S5) with the same NCHCF length of ~39 mm, checked in each case with a ruler scale. All five samples have similar transmission spectra with small variations in dips’ strength and position. It is noted that the variation between samples could be smaller if the length of NCHCF was more accurately controlled.

**Figure 8:**
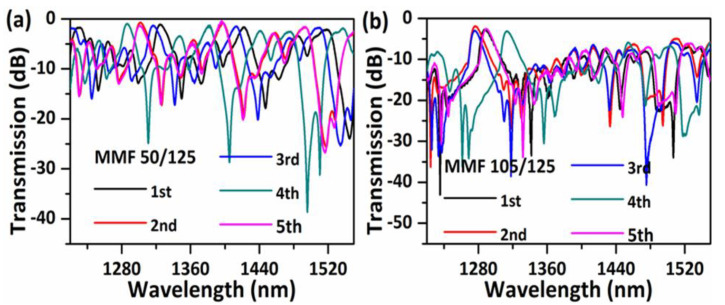
Measured transmission spectra of five sensor samples with an MMF length of ~39 mm for (**a**) MMF 50/125; and (**b**) 105/125.

As a comparison, ten samples of traditional SMS structures with the same length of ~39 mm, but with different core diameters of MMFs of 50/125 (core/cladding) and 105/125 were tested as shown in Figure 8 (five samples each). Variations in the measured transmission spectra of the NCHCF structure are much less than those of the SMS structure, indicating that our proposed NCHCF structure has a higher tolerance to the length change of the sensor head.

## 6. Conclusions

In conclusion, a novel fiber interferometer based on an NCHCF structure is proposed and investigated for the first time. FEM and BPM were used to analyze the mode field distributions and light propagation along the NCHCF structure. Clear evidence of the MMI mechanisms was observed in both simulation and experimental results. Both wavelength and spatial frequency spectral responses of the proposed sensor with different lengths of NCHCF were measured and discussed. The experimental results show that periodic transmission dips with large extinction ratio and wide FSR are excited due to MMI. ARROW guidance of a small portion of light is also observed. It is found that the transmission dips resulting from the MMI and ARROW effect have different sensitivities to strain and temperature; thus, it could be potentially used to monitor these two parameters with a single sensor head by using a characteristic matrix approach. It is also found that the sensor’s performance is independent of the surrounding RI, which facilitates its use in a broader range of applications. In addition, the proposed NCHCF structure is easy to fabricate, and has good reproducibility.
